# Supplementary material for: Real-time observation of the buildup of polaron in α-FAPbI3
Source: Nat Commun. 2023 Feb 17;14:917. doi: 10.1038/s41467-023-36652-4 (PMC9938110; doi:10.1038/s41467-023-36652-4)
Supplement: Supplementary file 1 — Supplementary Information [file 41467_2023_36652_MOESM1_ESM.pdf]

Supplementary Information for

Real-Time Observation of the Buildup of Polaron in α-$FAPbI_3$

Xingyu Yue,[1,2,3,#] Chunwei Wang,[1,3,4,#] Bo Zhang,[5,#] Zeyu Zhang,[1,2,3]*, Zhuang Xiong,[5,] Xinzhi Zu,[1,2,3] Zhengzheng Liu[1,2,3], Zhiping Hu,[2] George Omololu Odunmbaku[5], Yujie Zheng,[5] Kuan Sun[*5] and Juan Du*[1,2,3]

[1] *State Key Laboratory of High Field Laser Physics and CAS Center for Excellence in Ultra-intense Laser Science, Shanghai Institute of Optics and Fine Mechanics (SIOM), Chinese Academy of Sciences (CAS), Shanghai 201800, China*

[2] *School of Physics and Optoelectronic Engineering, Hangzhou Institute for Advanced Study, University of Chinese Academy of Sciences, Hangzhou, 310024, China*

[3] *Center of Materials Science and Optoelectronics Engineering, University of Chinese Academy of Sciences, Beijing 100049, China*

[4] *School of Physical Science and Technology, ShanghaiTech University, 100 Haike Road, Shanghai 201210, China*

[5] *MOE Key Laboratory of Low-grade Energy Utilization Technologies and Systems, CQU-NUS Renewable Energy Materials & Devices Joint Laboratory, School of Energy & Power Engineering, Chongqing University, Chongqing 400044, China*

[#] *These authors contribute equally to this work.*

**E-mail:* *zhangzeyu@ucas.ac.cn, kuan.sun@cqu.edu.cn, dujuan@siom.ac.cn*

This file includes

Supplementary Fig. S1 to Fig. S14

Supplementary Note S1 to Note S3

Supporting Information

Supplementary Figure S1

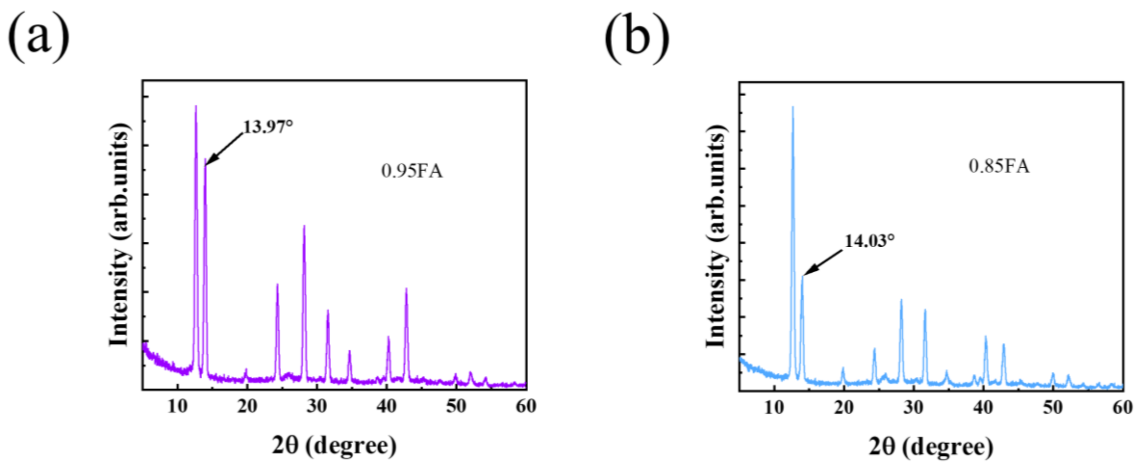

Figure S1 XRD characterizations of HOIPs. XRD data for (a) 0.85FA and (b) 0.95FA. Based on Bragg's law, since I^- is larger than Br^-, the diffraction angle of XRD peak increases with the increase of Br content.

Supplementary Figure S2

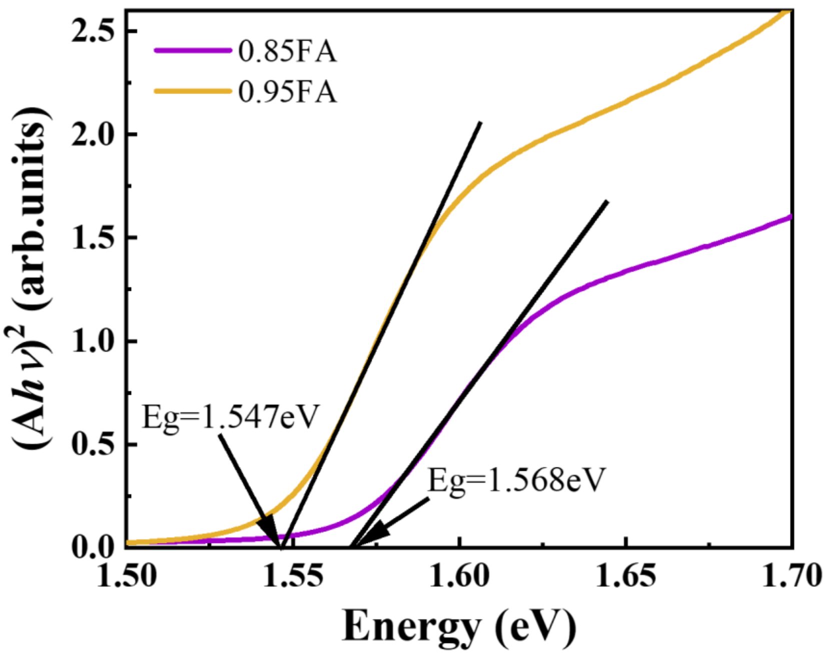

Figure S2 Absorption spectrum of 0.85FA and 0.95FA perovskites respectively.

Supplementary Figure S3

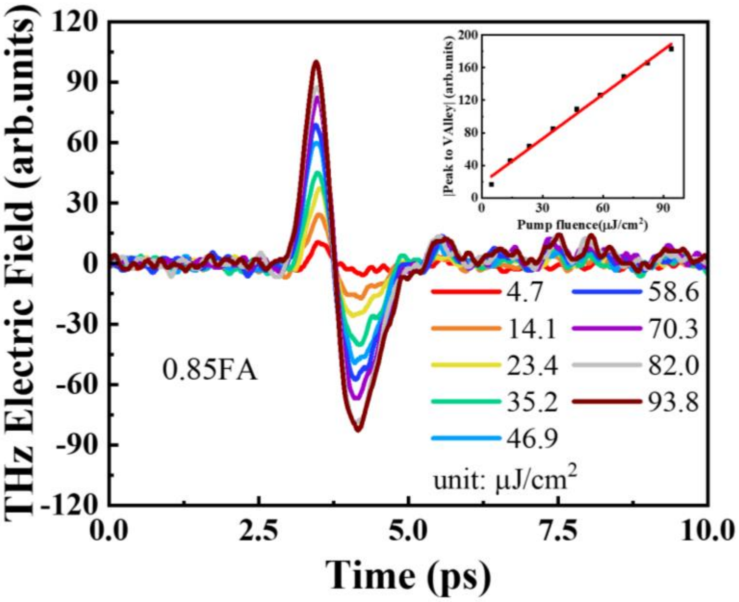

Figure S3 Terahertz (THz) emission due to ultrafast currents in HIOPs. THz time-domain emission spectra of 0.85FA measured with different excitation fluence at incident angle of 45 °; The inset shows pump fluence dependent peak-to-valley value of THz signals with 480nm laser excitation.

Supplementary Figure S4

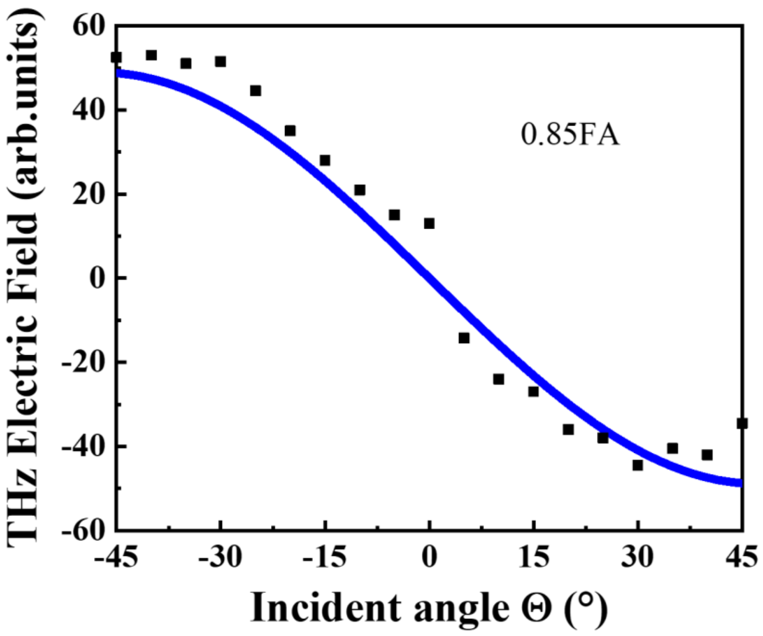

Figure S4 Incident angle θ dependent peak-to-valley value of THz signals with 480nm laser excitation, solid line is the theoretical curve calculated Eq. (1) in the text.

Supplementary Figure S5

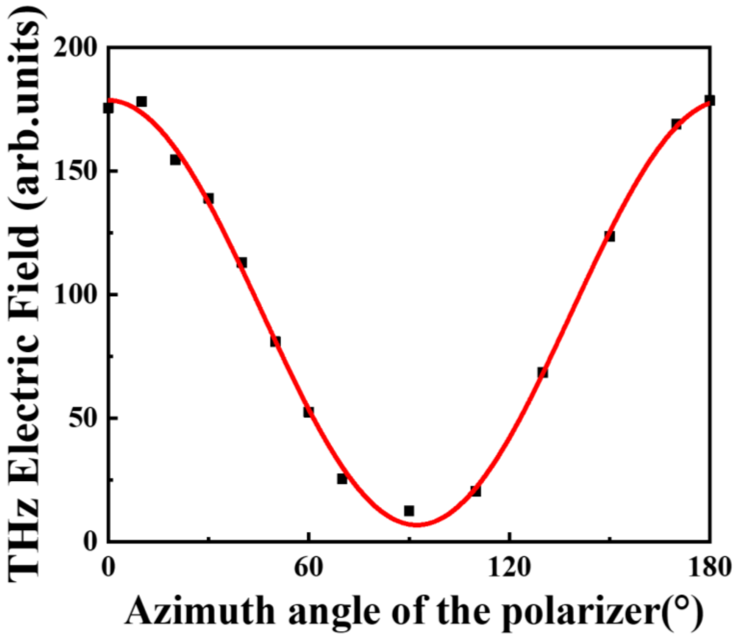

Figure S5 Polarization dependence of the emitted THz fields.

Supplementary Figure S6

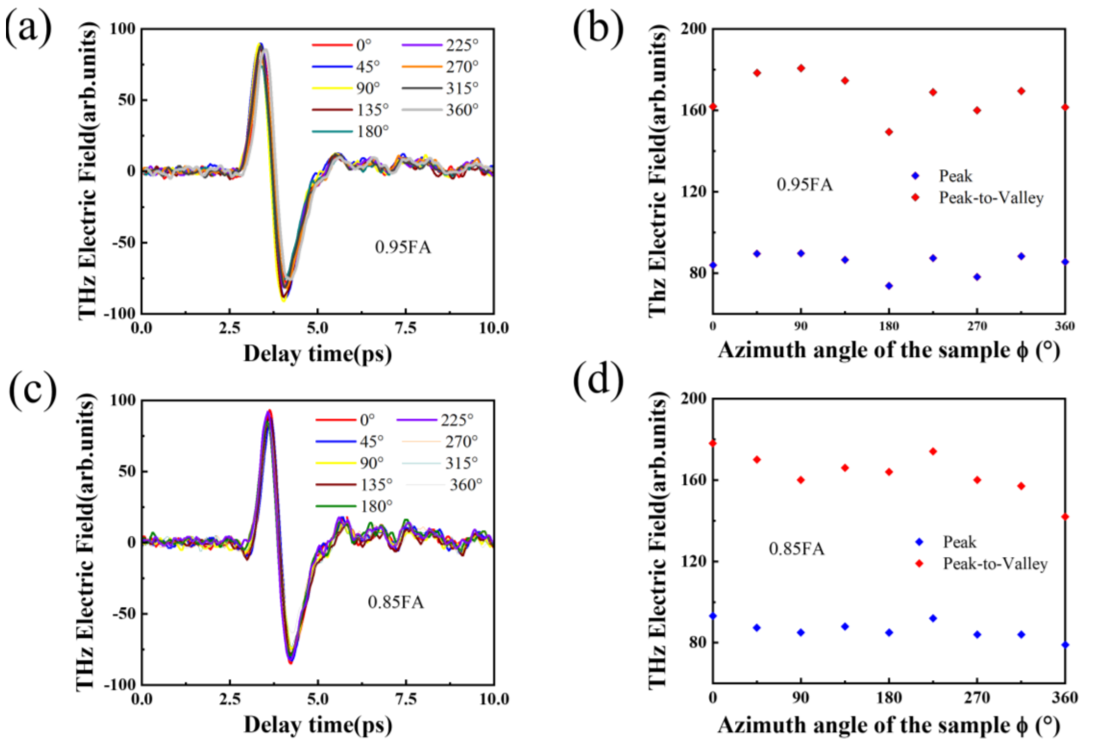

Figure S6 THz transients from 0.95FA (a-b) and 0.85FA (c-d) thin-films with different sample orientations. The emitted THz amplitude does not change upon changing the sample orientation shown for different φ angles.

Supplementary Figure S7

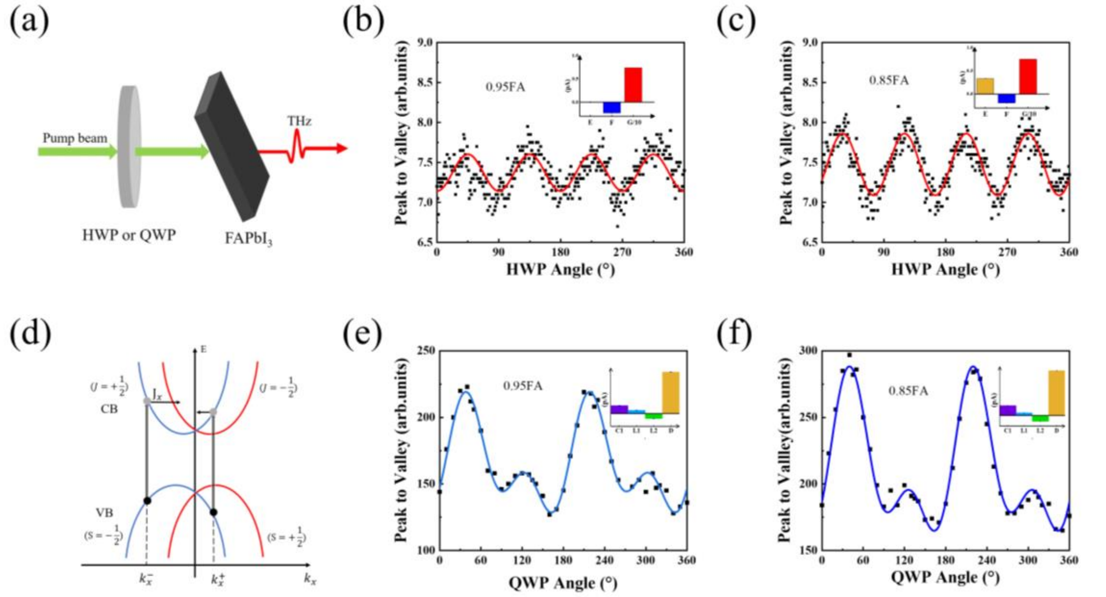

Figure S7 (a) Schematic diagram of the experimental setup for changing the polarization state of the pump beam with half-wave plate (HWP) and quarter-wave plate (QWP); Terahertz (THz) emission with half-wave plate in 0.95FA (b) and 0.85FA (c), inset in (b-c) are the fitting parameters C1, C2, L2, and D fitted from formula 2; (d) Schematic diagram of the continuum bands (VB and CB) having Rashba spin splitting, and related optical transitions with circular polarized light. The imbalance in momentum space causes the carriers to have a net velocity in a certain direction, which generates electric current; THz emission due to ultrafast photogalvanic (PGE) currents in 0.95FA (e) and 0.85FA (f), inset in (e-f) are the fitting parameters C_1, C_2, L_2, and D fitted from formula 2.

Supplementary Note S1

Fig. S7(a) shows the experimental setup for changing the polarization state of the pump light with quarter-wave plate and half-wave plate. From Fig.S7 a and Fig.S7 b, it can be seen that there are four equal peaks in the THz electric field intensity with the linear polarization direction of the pump light, which can be fitted by the following formula:

$$J(\beta) = Esin(4\beta) + Fcos(4\beta) + G \quad (1)$$

Here, β is the angle between the fast axis of the half-wave plate and the pump light polarization, E, F and G are constants. Since the half-wave plate can change the polarization direction of the linearly polarized light, this may be caused by the different absorption coefficients of s-light and p-light on the sample surface.

In HOIPs, the conduction band (CB) bottom consists of $J = {}^{1}/_{2}$states, whereas the valence band (VB) top has $S = {}^{1}/_{2}$ states[1]. Heavy atoms contribute to a giant spin−orbit coupling (SOC), leading to the splitting of the conduction band and valence band, which is called Rashba band splitting. Fig. S7 (d) depicts the splitting of the conduction band and valence band when the reverse pair forming is broken. According to the selection rule, the electron transition must be satisfied by $\Delta m_J = \pm 1$. When absorbing left (right) circular polarized light, the electron will be unbalanced in the momentum space, resulting in a certain direction of photocurrent, and different polarized light will lead to different directions of photocurrent. A quarter-wave plate is used to change the polarization of the pump light by rotating the angle, α between the fast axis of the QWP and the pump light polarization. Fi. S7(e) are THz emission signal peak-to-valley value of 0.95FA under 480nm optical excitation. The photocurrent can usually be expressed as[2]:

$$J(\alpha) = C_1 sin2\alpha + C_2 cos2\alpha + L_1 sin4\alpha + L_2 cos4\alpha + D \quad (2)$$

Here, C_1 is the CPGE current, C_2 is mainly due to misalignment between the fast axis of the λ/4 waveplate and the incident light polarization， and L_1 and L_2 are the photocurrent induced by linearly polarized light, which may be induced by linear

photogalvanic effect (LPGE) or photon drag effect， D is the photocurrent generated by the photo-Dember effect. Inset in Fig. 7(f) shows the fitting parameters C_1, L_2, and D fitted from the formula (3), the parameter D is much larger than the other parameters. Overall, the main mechanism of THz emission in HOIPs could come from the photo-Dember effect.

Supplementary Figure S8

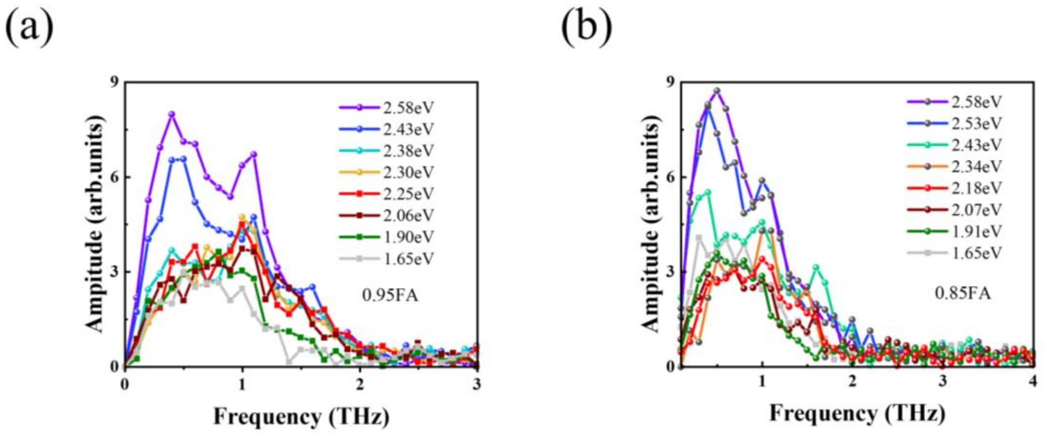

Figure S8. Amplitude spectrum of the emitted THz field for 0.95FA (a) and 0.85FA (b).

Supplementary Figure S9

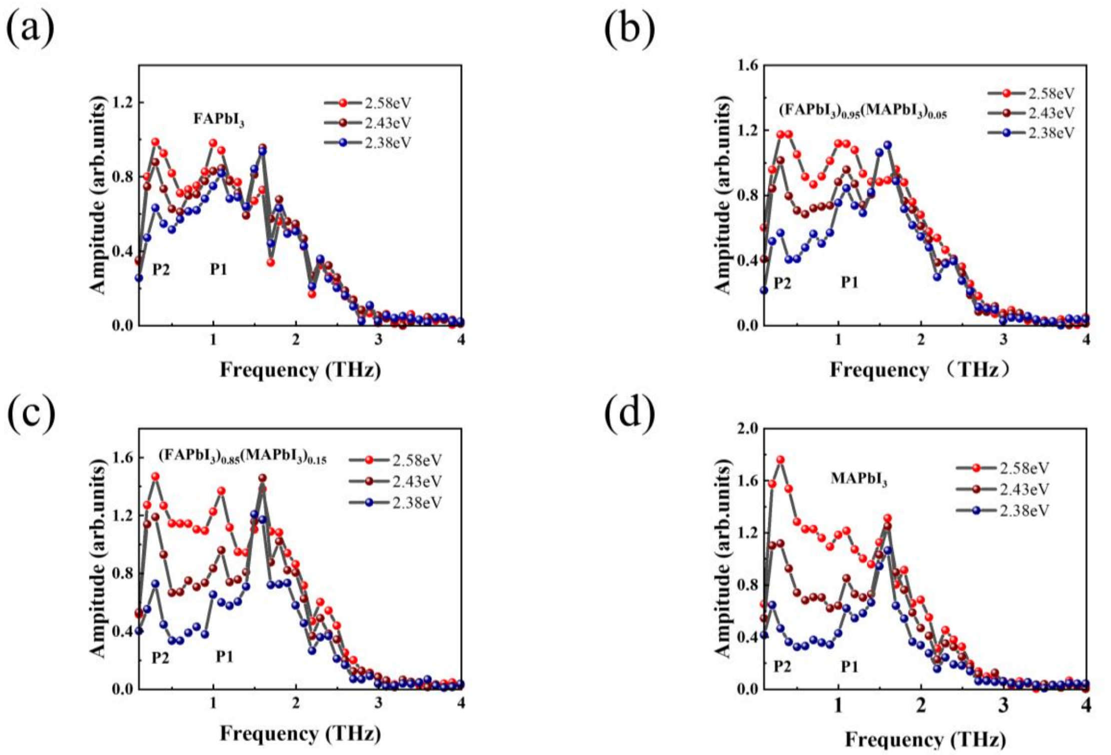

Figure S9. THz emission spectra of (a) $FAPbI_3$, (b) $(FAPbI_3)_{0.95}(MAPbI_3)_{0.05}$, (c) $(FAPbI_3)_{0.85}(MAPbI_3)_{0.15}$ and (d) $MAPbI_3$, measured with carrier concentration $n=2\times10^{18}$ cm^{-3} and different pump photon energy at 2.38eV, 2.43eV, 2.58eV respectively.

Supplementary Figure S10

(a)

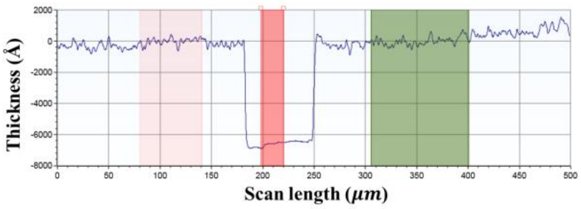

(b)

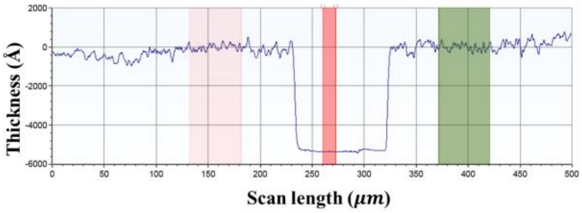

Figure S10. The thickness measured by the profilometry in (a) 0.95FA is ~658nm and which in (b) 0.85FA is ~534nm, respectively.

Supplementary Figure S11

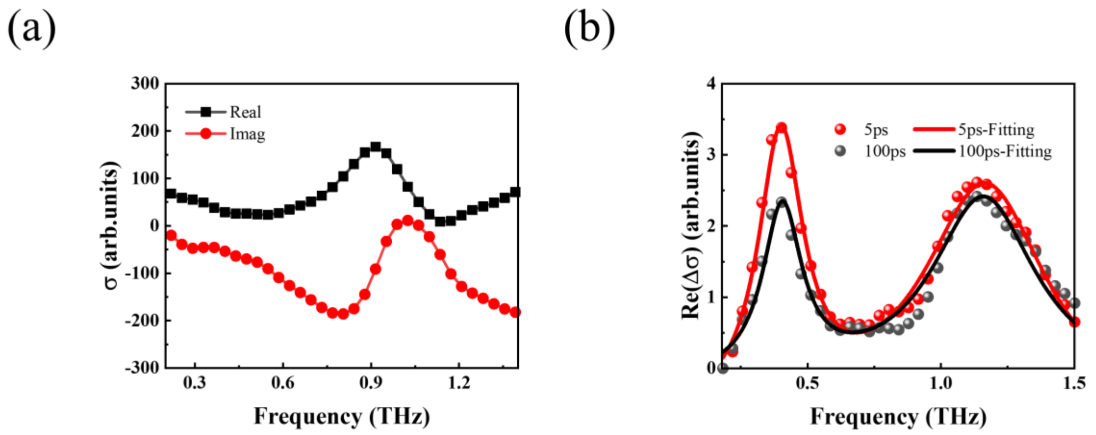

Figure S11. (a) The THz conductivity σ(ω) of unexcited 0.95FA; (b) Real part of transient optical conductivity $\Delta\sigma$ of 0.95FA with delay time of 5ps and 100ps with pump fluences of 70.3 μJcm-2 at 300 K.

We investigated the THz time domain spectroscopy (TDS) of the 0.95FA film, as shown in Fig. S11(a). The THz conductivity σ(ω) of the 0.95 FA film without optical pump show the phonon resonance mode at ~0.9 THz, which are assigned to angular distortion of the I-Pb-I phonon mode[3, 4]. Then, the optical pump-THz probes (OPTP) of the perovskite film could help to detect the photoinduced carrier coupled phonon mode in the 0.95FA film[4-6]. As shown in Fig. S11(b), transient optical conductivity acquired at 5ps and100ps decay times after the photoexcitation was measured. Two resonance peaks at ~0.4THz and ~1THz, which are corresponding to the A-site cation rotation polaron (P2) and inorganic sublattice vibration polaron (P1), respectively[4, 7]. The measured frequencies of the polaron modes are in consistent with our terahertz emission polaron modes (P1 and P2) in 0.95FA films. The peak intensity of two phonon modes at delay time of 100ps only decreases slightly compared with the one at delay time of 5ps. In contrast, the terahertz emission could present the dynamical anharmonic coupling between the photoinduced carriers and the phonon modes during the formation process of polarons, which have seldom reported by the OPTP measurements in HIOPs. Therefore, these measurements of TDS and OPTP could support our proposed anharmonic coupling model of the strong electron phonon coupling, which is experimentally proved by our terahertz emission spectroscopy.

Supplementary Figure S12

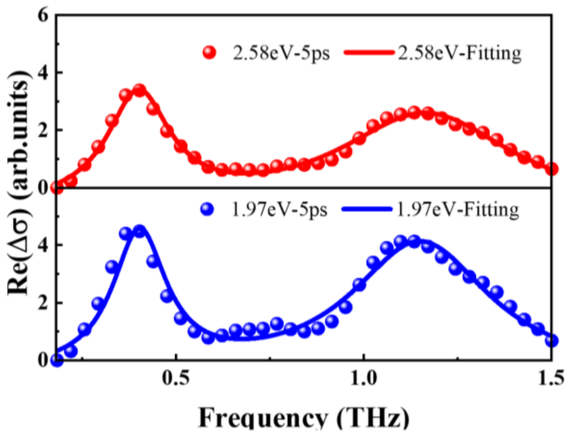

Figure S12. Real part of transient optical conductivity of 0.95FA with delay time of 5ps following photoexcitation at 2.58 eV and 1.97eV with pump fluences of 70.3 μJcm^{-2} at 300 K.

In OPTP measurement, the absorption peaks in the photo-induced THz conductivity are related to the intensity of phonon emission [8, 9]. As shown in Fig S12, we have used photon excitation below (630nm, 1.97 eV) and above (480nm, 2.58 eV) CB2 in the optical pump-THz probe (OPTP) spectroscopy to characterize the photo-induced THz conductivity. If the observed result is mainly due to the phonon emissions generated by the cooling of hot carriers associated with the excess energy dumping, different absorption intensity at P2 should be detected using photon excitation below and above CB2. However, as shown in Fig S12, the intensities of P2 are similar. Therefore, it is incomplete to use phonon emissions generated by hot carriers cooling to explain the THz emission enhancement of P2 observed using excitation above bandgap in our study.

Supplementary Figure S13

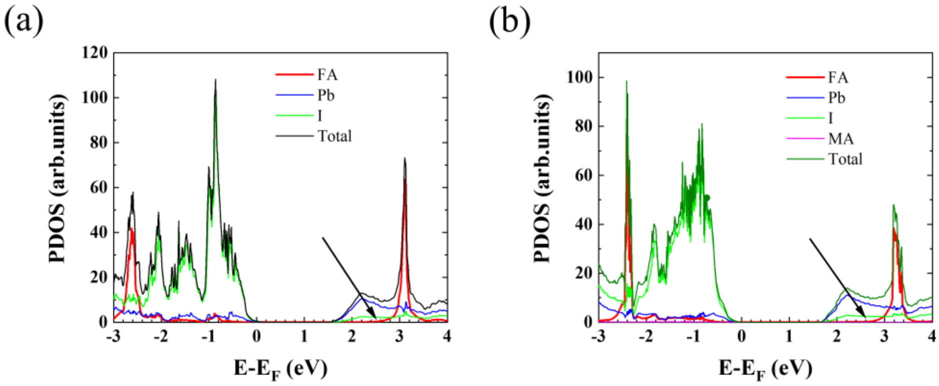

Fig.S13 (a) and (b) Projected density of state (PDOS) of $FAPbI_3$ and MA doped $FaPbI_3$, respectively

After the substitution of MA cation in $FAPbI_3$ (only one MA substitutes one FA in a $2 \times 2 \times 2$ supercell, i.e., $FA_{0.875}MA_{0.125}PbI_3$), the shapes of the energy curves shows that the upper conduction band of FA cations (red curve in Fig. S13(b)) shifts slightly. Hence, with the existence of MA, the transition process could be similar with the condition without MA substitution.

Supplementary Figure S14

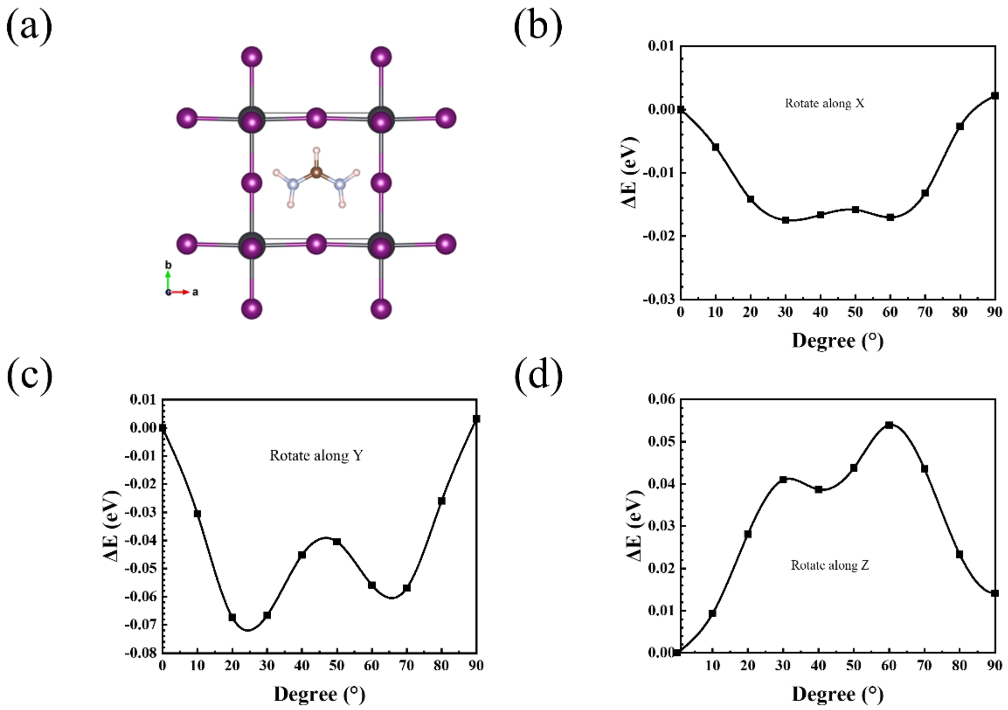

Figure S14. (a) Top view of initial atomic structure of FAPI3. The inset (left-down) shows the direction of a (X) b (Y), and c (Z). (b), (c), and (d) Potential energy curves for rotations of FA along X, Y, and Z axis, respectively.

We checked the electronic energy curves (Fig. S14) associated with the orientation of the FA molecule by DFT calculation. Fig. S14 (b-d) shows the potential energy difference (ΔE) curves of FA rotation along X, Y, and Z axis. When FA rotates or vibrates in a certain direction, the energy difference of the system is less than 0.1eV (depending on the degree of the rotation). This indicates that the orientation of FA cations does not significantly affect the total electronic energy of $FAPbI_3$.

Supplementary note S2

In the transition model, we assume that electrons are vertically excited from the VB to CB1 and CB2, where CB2 contains the energy level of the FA cations. To support this transition model, we checked the electronic energy (Fig. S14) curves and electronic structure (Fig. S13) associated with the orientation of the FA molecule. Fig. S14 (b-d) shows the potential energy difference (Δ E) curves of FA rotation along X, Y, or Z axis. When FA rotates or vibrates in a certain direction, the energy difference of the system is less than 0.1eV (Depending on the degree of the rotation). This indicates that the orientation of FA cations does not significantly affect the total electronic energy of $FAPbI_3$, hence, the interactions between FA and Pb-I framework are weak.

Supplementary note S3

The density functional theory (DFT) calculation: The density functional theory (DFT) calculations were performed using the VASP code[10, 11] using the projector-augmented plane wave (PAW) approach and a kinetic energy cutoff of 500 eV. The generalized gradient approximation (GGA) with Perdew-Burke-Ernzerhof (PBE) exchange-correlation functional[12] was employed to perform all calculations and Grimme's D3[13] correction was used to consider the van der Waals (vdW) interactions. The cubic-phase FAPbI3 was selected and a $2\times2\times2$ supercell was used to model the MA doping. The potential energy curve is obtained every $10°$ rotation of FA. The geometry of FA was fixed, and the PbI6 inorganic frameworks and lattice constants were optimized to reproduce the symmetry lowering associated with the FA rotation and the coupling between Fa and the dynamic notions of the PbI6 framework.

THz time domain spectroscopy (THz-TDS): Broadband THz-TDS was carried out on the 0.95FA. The fundamental laser pulse with wavelength at 800 nm is generated by a Ti: sapphire amplifier. The fundamental laser beam was focused on ZnTe to generate THz emission. And the fundamental laser beam was modulated by an optical chopper. Measure the THz transmission signal of the resulting THz as it passes through the sample and through the substrate.

Optical pump/THz electromagnetic probe (OPTP) spectroscopy:
The OPTP system is driven by a Ti:sapphire fs amplifier laser pulse of ~35 fs duration with a repetition rate of 1 kHz. The fundamental laser pulse is divided into three beams, a pump beam, a THz generate beam, and the other beam is used as probe light to detect THz signal emitted by the sample. The pump beam was modulated by an optical chopper. A time-delay line is used to vary the optical path of the pump beam and the THz generated beam. The THz transmission signal of the sample under different time delay after optical pumping was measured.

Supplementary References

1. Li, J. & Haney, P.M. Circular photogalvanic effect in organometal halide perovskite CH3NH3PbI3. *Applied physics letters* **109**, 193903 (2016).
2. Liu, X. et al. Circular photogalvanic spectroscopy of Rashba splitting in 2D hybrid organic–inorganic perovskite multiple quantum wells. *Nature communications* **11**, 1-8 (2020).
3. Guzelturk, B. et al. Terahertz emission from hybrid perovskites driven by ultrafast charge separation and strong electron–phonon coupling. *Advanced Materials* **30**, 1704737 (2018).
4. Jin, Z. et al. Photoinduced large polaron transport and dynamics in organic–inorganic hybrid lead halide perovskite with terahertz probes. *Light: Science & Applications* **11**, 1-12 (2022).
5. Cinquanta, E. et al. Ultrafast THz probe of photoinduced polarons in lead-halide perovskites. *Physical review letters* **122**, 166601 (2019).

6. Sarkar, S. et al. Terahertz spectroscopic probe of hot electron and hole transfer from colloidal CsPbBr3 perovskite nanocrystals. *Nano letters* **17**, 5402-5407 (2017).
7. Selig, O. et al. Organic cation rotation and immobilization in pure and mixed methylammonium lead-halide perovskites. *Journal of the American Chemical Society* **139**, 4068-4074 (2017).
8. Ulbricht, R., Hendry, E., Shan, J., Heinz, T.F. & Bonn, M. Carrier dynamics in semiconductors studied with time-resolved terahertz spectroscopy. *Reviews of Modern Physics* **83**, 543 (2011).
9. Zhao, D. & Chia, E.E. Free carrier, exciton, and phonon dynamics in lead‐halide perovskites studied with ultrafast terahertz spectroscopy. *Advanced Optical Materials* **8**, 1900783 (2020).
10. Kresse, G. & Furthmuller Efficient Iterative Schemes for ab Initio Total-Energy Calculations Using a Plane-Wave Basis Set. *Phys. Rev. B* **54**, 11169-11186 (1996).
11. Kresse, G. & Furthmuller, J. Efficiency of ab-Initio Total Energy Calculations for Metals and Semiconductors Using a Plane-Wave Basis Set *Comput. Mater. Sci.* **6**, 15-50 (1996).
12. Perdew, J.P., Burke, K. & Ernzerhof, M. Generalized Gradient Approximation Made Simple. *Phys. Rev. Lett.* **77**, 3865-3868 (1996).
13. Grimme, S., Antony, J., Ehrlich, S. & Krieg, H. A Consistent and Accurate ab Initio Parametrization of Density Functional Dispersion Correction (DFT-D) for the 94 Elements H-Pu. *J. Chem. Phys.* **132**, 154104 (2010).
